# Efficacy of the Fumigant Ethanedinitrile to Control the Ham Mite, *Tyrophagus putrescentiae* (Schrank) (Sarcoptiformes: Acaridae), and Its Sorption on Dry-Cured Ham

**DOI:** 10.3390/insects16010007

**Published:** 2024-12-27

**Authors:** Jacqueline M. Maille, Wes Schilling, Thomas W. Phillips

**Affiliations:** 1 Department of Entomology, Kansas State University, 123 W. Waters Hall, Manhattan, KS 66506, USA; jmaille@ksu.edu; 2 Department of Food Science, Nutrition and Health Promotion, Box 9800 Room 203 Bost Extension Center, Starkville, MS 39762, USA; schilling@foodscience.msstate.edu

**Keywords:** mold mite, ham mite, cheese mite, country ham, methyl bromide alternative

## Abstract

Management of stored-product pests has historically relied on fumigation when pest populations become large. However, the ban of the fumigant methyl bromide and the ineffectiveness of other pesticides stress the need for alternative fumigants. Therefore, laboratory studies were conducted to evaluate the efficacy of ethanedinitrile (EDN) against different life stages of the mite *Tyrophagus putrescentiae* and to determine the sorption and desorption of EDN by dry-cured ham meat. The results showed that eggs were the most tolerant life stage to EDN fumigation, with an estimated LC_50_ of 0.6 mg/L. *Tyrophagus putrescentiae* mixed life-stage colonies were controlled at 1.3 mg/L, and less than 0.05% of the population survived following treatment with 0.6 mg/L within 24 h at 25 °C. The free-headspace concentrations of EDN in fumigation chambers containing ham decreased by 97% of the initial concentrations applied (2.6 and 4.8 mg/L) after the 24 h fumigation period. The EDN sorption in ham followed the first-order kinetics, with half-life values of 5.0 and 4.9 h for 2.6 and 4.8 mg/L, respectively. The percentage losses of EDN per hour were calculated to be 12.8 and 13.2% at 2.6 and 4.8 mg/L, respectively. Our study indicates that EDN controls *T. putrescentiae* in the laboratory.

## Simple Summary

Stored-product pests are usually controlled with fumigation, but one common fumigant, methyl bromide, has been banned, and other pesticides have been found to not be as effective. So, we studied a new fumigant called ethanedinitrile (EDN) to see if it could control a mite, *Tyrophagus putrescentiae* and how the fumigant interacts with dry-cured ham. The study showed that *T. putrescentiae* eggs were the hardest to kill, with an effective concentration of 0.6 mg/L. At a concentration of 1.3 mg/L, the entire population was killed, and at 0.6 mg/L, less than 0.05% survived after 24 h at 25 °C. When EDN was used in chambers with ham, 97% of it was absorbed by the ham after 24 h. The absorption rate followed a predictable pattern, with half of the EDN absorbed in 5 h at the lower concentration (2.6 mg/L) and about the same time for the higher concentration (4.8 mg/L). EDN was lost at rates of 12.8% and 13.2% per hour, depending on the concentration. This study shows that EDN can be an effective way to control *T. putrescentiae* in the lab, which could help manage these pests in the future.

## 1. Introduction

*Tyrophagus putrescentiae* (Schrank) (Sarcoptiformes: Acaridae) is also known by the common names mold mite, ham mite, or copra mite. This species is a pest of over 140 commodities [1], many of which contain high-protein, high-fat, and high moisture, such as dry-cured meats, artisanal cheeses [2,3], semi-moist pet foods [4,5,6], and dried fruits [7]. *T. putrescentiae* infestations usually occur on the surface of a commodity. However, penetration into the commodity can occur [8], causing considerable economic damage because the commodity may be discarded. In addition, it has been recorded that the mite can cross-contaminate commodities with spores of fungi that produce aflatoxins [9]. Due to their small size, with females ranging from 320 to 420 µm and males ranging from 280 to 350 µm long, infestations of these mites are not readily observed by stored product managers and commonly persist until mite dust forms on the surface of the product. The progression of *T. putrescentiae* life stages are an egg, a six-legged larva, a protonymph, a tritonymph, and a reproductive adult [10]. *Tyrophagus putrescentiae* females begin laying eggs within 24 h of mating and can oviposit up to 500 eggs in a lifetime [11], combined with a generation of mites occurring every 8–53 days depending on the commodity and environmental conditions [12]. The capacity for exponential population growth combined with its minute appearance, their ability to spread aflatoxins, and the nil-tolerance of mite infestations on U.S. dry-cured ham products [13] has led to significant product losses for dry-cured ham plants.

Management strategies to combat *T. putrescentiae* are being adapted using specialized sanitation measures, traps for detection and monitoring [4,14,15], temperature management [16], manipulated lighting [15], food-safe coatings [17,18,19], food-grade protective netting [20], residual pesticides [21], and fumigants [22,23,24,25]. All of these strategies, separately or in combination, have been utilized to eliminate or suppress mite populations in laboratory or semi-field settings. Fumigation is the most favored control strategy, as it is ideal for penetrating products and areas that are otherwise inaccessible by conventional topical pesticides. In addition, fumigants create a swift knockdown of a pest population with minimal chemical interaction with the commercial product. Many fumigants are available but have been proven to be unsatisfactory. Methyl bromide is a broad-spectrum fumigant that was banned for most applications due to its ozone-depleting properties and is only usable in quarantine situations [26,27,28]. Alternatively, phosphine is effective at controlling *T. putrescentiae*, but it is corrosive to most metals and is therefore unsatisfactory for use in dry-cured ham aging facilities with electrical machines, lighting, and other circuitry [29]. Another fumigant is sulfuryl fluoride, but it is ineffective at controlling *T. putrescentiae* eggs at room temperature conditions within allowed concentrations [30]. Ethanedinitrile has been shown to exert significant toxicity against a wide range of stored-product pests [31,32,33,34]. However, as far as we know, no studies have been reported to evaluate EDN toxicity against *T. putrescentiae*.

Twenty-five years ago, O’Brien et al. (1999) patented ethanedinitrile (EDN) as a broad-spectrum fumigant for use against insects, arachnids, nematodes, bacteria, molds, and rodents on a variety of commodities, including but not limited to grain, seeds, meats, fruit, vegetables, timber, and plants [35]. The −21 °C boiling point of EDN with a 515 kPa vapor pressure at 21 °C makes EDN an ideal fumigant [36]. The United States National Institute for Occupational Safety and Health (NIOSH) has a recommended exposure limit (REL) of 10 ppm (*v*/*v*) for EDN, which compares favorably to both methyl bromide (5 ppm) and phosphine (0.3 ppm). Another benefit of this compound is that it is a non-ozone-depleting substance [37]. EDN can break down chemically to hydrogen cyanide and other cyanide-bound molecules, and thus its commercial use will require research on the effectiveness of extensive safety measures to avoid human exposure. To the best of our knowledge, this is the first report to study EDN efficacy against *T. putrescentiae*. Consequently, the current study aimed to determine the effectiveness of EDN against the life stages of *T. putrescentiae* and verify these findings by applying the recommended concentration of EDN to completely control the mixed life-stages culture. Attention to adequate concentrations of a fumigant needed for a good kill must be considered, along with the reduced gas concentration that can happen due to the presence of the commodity being protected, referred to as sorption. We therefore studied the sorption and desorption dynamics of EDN in the presence of cured ham meat at two applications concentrations of 2.6 and 4.8 mg/L at 25 °C for 24 h of fumigation.

## 2. Materials and Methods

### 2.1. Ham Mite Cultures

*Tyrophagus putrescentiae* laboratory cultures have been maintained in the Department of Entomology at Kansas State University since 2008 when they were derived from an active field infestation. Mite-rearing methods were followed according to earlier work in our lab [16] whereby approximate 1 L glass wide-mouthed Ball^®^ Mason jars (Westminster, CO, USA) (85 mm in diameter; 160 mm in height) containing mite diet and then sealed with a labeled P8 Fisherbrand 9.0 cm diameter filter paper and the metal lid screw-on ring. The mite diet was composed of 75 g of a ground commercial dog food consisting of a minimum of 23% crude protein, 12% crude fat and 4% crude fiber at a 14% moisture content, and 475 mL of water that was combined and heated for six minutes in a microwave (General Electric Co., Boston, MA, USA, 0.95 KW). The mixture was then blended smoothly in a 120 V Hamilton Beach Blender©, and then the mixture was spooned into a one-liter beaker, where it was heated on a hotplate until boiling. Agar (ICN Biomedicals Inc., Costa Mesa, DE, USA), alphacel (ICN Biomedicals Inc.), yeast (MP Biomedical LLC, Solon, OH, USA), and Vanderzant modification vitamin mix for insect diets (MP Biomedicals LLC) at a 5:5:5:5 g ratio, respectively, and 25 mL of glycerol (Fisher Scientific, Pittsburgh, PA, USA) were added to the mixture and brought back to a boil. Then, 5 mL of 15% methyl-p hydroxybenzoate in 95% ethanol (ICN Biomedicals LLC) was added and heated for an additional 10 min. The cooked mixture was portioned evenly and combined with 14 g of the whole-kibble commercial dog food in mite-rearing jars. Approximately 300 mL of cooked mite diet was poured into the glass jars. Approximately 500 mixed life-stage mites were introduced from a previous healthy culture after the diets had been cooled to 25 °C, and then they were placed in a rearing incubator (Percival Scientific, Inc., Perry, IA, USA) at 25.5 ± 2.5 °C and 70–80% R.H. in total darkness.

### 2.2. Mite Life-Stage Separation

Healthy protonymphs, tritonymphs, and adults were classified as mobiles and selected using a one bristle Taklon^®^ paintbrush, no. 2 (Toray Chemical Co., Osaka, Japan) from the mass of mites walking on the underside of the filter paper lid. Many of the mites selected were gravid adult females due to their large size. The twenty selected mites were transferred individually into a 1.8 mL glass vial containing a 27 mm^3^ piece of 6-month-aged country ham (Tripp Country Hams, Brownsville, TN, USA). The vials were labeled, covered with 30 μm mite mesh, and capped with a plastic ventilated lid. Vials were stored at 22.5 ± 2.5 °C and 70 ± 2% R.H. under total darkness for up to 24 h before fumigation.

Mite eggs were collected by first selecting 50 healthy large mites, presumably mostly gravid adult females, from the filter paper lid of the glass vial. These mobiles were then placed in a 118 mL glass jar with ~20 g of cooked mite diet and covered with a labeled P5 Fisherbrand 7.0 cm diameter filter paper (Fisher Scientific, Pittsburgh, PA, USA) and a metal ring. The adults were left for 48 h in a double water bath of ambient temperature being ~25 °C and 70% R.H. in total darkness. Ten mite eggs were then individually removed from the diet using a one-bristle paintbrush and placed onto double-sided sticky tape that was taped to a 1 cm^2^ piece of black construction paper that was then placed into a glass vial that contained a 27 mm^3^ piece of ham. The egg sheets of 10 eggs were placed into labeled 1.8 mL vials covered with 30 μm mesh screens with a plastic ventilated shelf vial lid. Vials were stored at 22.5 ± 2.5 °C and 70 ± 2% R.H. for up to 3 h until fumigation. All experiments described here were conducted between May and November 2018.

### 2.3. Fumigant Concentration–Mortality Assays

Ethanedinitrile (the commercial product, Cyanogen, ≥99.90%) was supplied by Draslovka Services Pty Ltd., Sydney, Australia. Two-liter glass jars, 23 cm tall and 10 cm wide, served as fumigation chambers that were each loaded with four 1.8 mL vials, each containing a 27 mm^3^ piece of 6-month-aged country ham (Brownsville, TN, USA). Two vials in each jar contained 20 mobile mites to evaluate EDN toxicity to mobiles, while another two vials each had ten eggs placed on a double-sided tape. A 2 mL glass vial of water was placed inside each fumigation jar to standardize and maintain internal humidity. The mites in each fumigation jar represented one treatment unit for fumigation or the non-fumigated controls. The jars were sealed with a gas-tight screw-on metal lid that was modified with a copper-tube gas introduction port 7 mm wide × 15 mm tall sealed to an opening in the middle of the jar lid. Three jars were fumigated at each of 8–12 target EDN concentrations for 24 h. Target gas concentrations were selected within the range of concentrations tested in earlier work with postharvest insect pests [31,34,38,39]. Mites were exposed to 0, 0.27, 0.31, 0.36, 0.38, 0.40, 0.42, 0.44, 0.48, 0.53, and 0.62 mg/L; and 0, 0.2, 0.26, 0.27, 0.31, 0.36, 0.44, and 0.62 mg/L for mobile and egg sheets, respectively. To achieve the target concentrations, the desired volume of gas per target concentration was calculated based on the volume of the fumigation jar. Before fumigation, pure EDN was drawn from the cylinder into a 0.5 L Tedlar^®^ (polyvinyl fluoride) gas sampling bag (CEL Scientific Corp., Cerritos, CA, USA). The desired volume of EDN to be added to each fumigation jar was then taken from the Tedlar bag using a gas-tight syringe (Hamilton, Reno, NV, USA) and injected through the gas-tight septum after removing an equal volume of air from the jar. Fumigations were held for 24 h at 25 ± 1 °C with 70% R.H. and a 16 h photoperiod. Before vial removal, fumigation jars were opened and aerated for one hour. Vials were then placed into a desiccator containing a saturated NaCl solution at room temperature 22.5 ± 2.5 °C and 70 ± 2% R.H. for a recovery period of 72 h for mobiles and 168 h for eggs. Mortality of mobiles was determined by observing their inability to move, and eggs were counted as dead by failing to hatch into larvae using an Olympus S2x10 stereoscope (Olympus Life Sciences, Waltham, MA, USA) at 63× magnification. Any mobile stage that did not physically react following a 30 s observation period at the end of the 72 h was considered dead. The concentration–response assays of EDN were analyzed using the proportion of mortality, and the concentration of the gas using probit regression in SAS Studio 3.8^®^ software [40] and the natural death found in controls were used as a correction factor for each life stage.

### 2.4. Fumigant Detection and Quantification

Fumigant detection followed the methods of Ramadan et al. [32]. Concentrations of EDN present in the fumigation jars were measured using a coupled gas chromatograph–mass spectrometer, GC-MS (Shimadzu17A QP5050A, Shimadzu Scientific Instruments, Kyoto, Japan) in the selected ion monitoring (SIM) mode with the electron impact (EI) ionization at 1.3 kV. The GC was equipped with a J&W Scientific DB-1MS UI (30.0 mm length × 0.250 mm I.D × 0.25 µm film thickness) capillary column (Agilent Technologies, Santa Clara, CA, USA). The carrier gas was ultra-pure helium at a flow rate of 1.1 mL/min. The oven temperature was isothermal and set at 150 °C. The injection port was set up for split injection at 250 °C with the MS detector transfer line at the same temperature. The quantification of EDN was carried out in the SIM mode for the EDN molecular ion 52 *m*/*z*. The concentration of EDN was calculated for each treatment based on an external standard curve we generated just before each fumigation experiment. The standard curve was achieved by diluting a precise volume of the 100% EDN into a known volume of air in a Tedlar bag. Injections of this standard gas at volumes of 25, 20, 15, 10, and 5 μL were analyzed by GC-MS. The area of the peak resulting from an injection of 15 μL was established to be equivalent to the standard concentration, while the other injection volumes represented proportionally smaller or larger amounts of EDN to generate the standard curve. The EDN concentration in each fumigation jar was determined with quantitative GC-MS, using a headspace sample of 15 µL at the beginning and the end of the 24 h fumigation period. The average concentration for each jar was calculated from these two measurements and assigned to the mite mortality data from the respective jar, allowing us to confirm that target concentrations were achieved.

### 2.5. Mixed Life-Stage Fumigation

Plugs of food bait were altered from Amoah et al. 2017 [15] and made using methods described by Maille et al. [25]. The food plugs were placed in the center of a 118 mL glass jar. A Taklon^®^ No. 2 paintbrush was used to gently brush the underside of the culture’s filter paper lid into a tared 11 mL glass vial until 55 mg of mites (~7000 mites) were accumulated by the measurement of an Accuris Instruments Analytical Series W3100A-210 Balance (Marshall Scientific, Hampton, NH, USA). The vial was emptied onto the food plug, and the jar was secured with 30 µm mite mesh, a rubber band, and a metal ring. The rubber band secured the mite mesh above the metal ring to ensure water would not seep into the mite mesh. Jars of mixed life stages were placed into a soapy water bath and held for up to 12 h at 22.5 ± 2.5 °C and 70 ± 2% R.H. to allow adult mites to acclimate to their new environment and lay eggs on the food bait to ensure a mixed life stage was present for fumigation. Following this, the jars were placed into a two-liter wide-mouthed glass jar containing an 11 mL vial with 2 mL of water. The jars were sealed with a gas-tight screw-on metal lid that was modified with a gas introduction port and then fumigated at target concentrations of 0, 0.6, and 1.8 mg/L by injecting 0, 54, or 27 μL of EDN, following the previously stated mortality assay methods. Jars were placed in an incubator set at 25 ± 1 °C with 70% RH and a 16 h photoperiod for 24 h. After 24 h, jars were vented in the fume hood for one hour. Colonies of mixed life stages were removed from fumigation jars and placed into a desiccator at room temperature, 22.5 ± 2.5 °C, and 70 ± 2% RH for a recovery period of 72 h to count the surviving mobiles. After mobile survival was counted, jars of mixed life stages were placed into a 30 °C water bath, and a 5 × 1 cm piece of paper was held close to the food plug to remove the mobiles. The jars of mixed life stages were placed back into the desiccator at previously described conditions until 168 h after the fumigation to count for egg hatch. Fumigation of mixed life-stage groups was replicated five times.

### 2.6. Sorption and Desorption of EDN on Dry-Cured Country Ham

The sorption of EDN at concentrations of 2.6 and 4.8 mg/L was conducted according to Ramadan et al. [32], using 2 L Ball Mason wide-mouth jars (Ball Corp., Broomfield, CO, USA) as fumigation chambers. There were three replicate jars (n = 3) set up for each of the two EDN treatments. Two hundred grams of dry-cured country ham cut into a 10 cm × 10 cm × 20 cm block (Tripp Country Hams, Brownsville, TN, USA) was transferred into each of the fumigation chambers. All fumigation chambers were then tightly closed with a gas-tight screw-on metal lid equipped with a gas introduction port for gas injection. Gas was applied at 0, 117, or 192 μL of EDN into the injection port of the fumigation jars. Glass beads (spherical 14 mm dia., Hobby Lobby, Inc., Oklahoma City, OK, USA) were put into one fumigation chamber for each concentration to displace the same volume of space as displaced by the 200 g of ham cubes. The glass beads served as an inert surface to determine if any sorption (loss of concentration) of EDN associated with the ham in the fumigation chambers was also found in the jars with relatively inert surfaces of the glass beads. Also, an empty fumigation chamber was used for the construction of the standard calibration curve at a concentration of 2.16 mg/L to calculate the concentrations of EDN. The fumigation chambers for the sorption–desorption experiments were sealed using the metal lids, and EDN (99.9%) was taken from the cylinder into a Tedlar gas sampling bag (CEL Scientific Corp., Los Angeles, CA, USA). The desired volume of EDN for each concentration was calculated depending on the remaining space in the fumigation chambers after 200 g of ham meat was added. The desired volume of EDN was then taken and injected through the gas introduction port into the fumigation chambers using the gas-tight syringe (Hamilton, Reno, NV, USA). All fumigation chambers were kept at 25 °C in the incubator during the experiment. The free-headspace concentrations of EDN in the fumigation chambers were determined after 10 min, and then, at 1, 5, 8, and 24 h, using the GC-MS with the methods described above. For desorption analysis at 24 h, all fumigation chambers were opened after 24 h and ventilated in the fume hood for 1 h. The fumigation chambers were then re-sealed using the metal lid with a new gas septum applied to the injection port. All fumigation chambers were stored in the incubator at 25 °C, and the free-headspace concentrations of EDN, if any were detected following ventilation and re-sealing, were measured after 0 h, 4 h, 8 h, 24 h, and 1 week after sealing. The standard calibration curve chamber was stored until the end of the desorption experiment. The calibration curve was generated by injecting different volumes of 25, 20, 15, 10, and 5 μL from the held calibration chamber into the GC-MS. Then 15 μL were taken from the headspace of each fumigation chamber at each time interval using a 25 µL gastight syringe which was considered as the 2.16 mg/L concentration in the case of the standard curve. A standard curve was generated at each sample time interval to achieve accurate measuring of EDN concentrations. Three injections from each fumigation chamber and the calibration curve chamber were made and the average was used for calculations. The standard curve with a linear equation (R^2^ > 0.999) was calculated and used to estimate the concentration of EDN in the fumigation chamber from the area integrated under the GC peak for a given EDN sample. The initial concentrations of EDN at the beginning, C_0_ (10 min after injection), were calculated based on the remaining volume in the fumigation chamber after adding the ham according to this equation Va = C_0_ * S/C_s_, where (V_a_) is the volume of EDN to be added to each fumigation chamber, (S) is the remaining space in each fumigation chamber, and (C_s_) is the concentration of the EDN source.

The free-headspace concentrations of EDN in the fumigation chambers containing the ham meat were found to decrease over time following the first-order kinetics equation (C_t_ = C_0_e^−kt^) because an exponential decay model was found to apply with a higher correlation coefficient (R^2^), where C_t_ is the EDN concentration at the time t, C_0_ is the initial concentration of EDN, and k is the sorption rate constant (mg/time unit). The half-life values (t_1/2_) were calculated using the equation: t_1/2_ = ln 2/k. Also, the percentage loss of EDN concentration per hour was calculated following the equation of 100(1 − e^−k^). The sorption rate of EDN (mg/kg) by ham meat was calculated for both application concentrations of 2.6 and 4.8 mg/L.

## 3. Results

### 3.1. Fumigant Toxicity Assays

**Figure 1:**
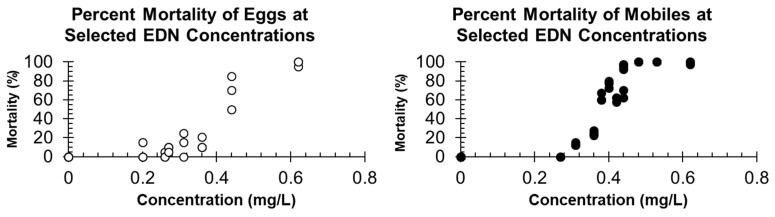
The mortality of *T. putrescentiae* eggs (**left**) and mobile life stages (**right**) after fumigation to a selected range of EDN concentrations during a 24 h fumigation (n = 3).

The percent mortality for given average concentrations of EDN fumigated for 24 h is shown in Figure 1. Natural death in individuals occurred in the controls only 10/961 (0.01%) and 5/608 (0.008%) for mobiles and eggs, respectively. These natural death proportions were used as a correction factor for the respective Probit analyses.

The probit regression analyses of EDN for eggs and mobile stages are reported in Table 1. The Pearson goodness-of-fit (χ^2^) test showed that the fit differences between the calculated probit regression model to the observed data were significant (*p* < 0.05) for both probit model regressions, and thus a poor representation of the data. Since the *p*-values for the tests were low, variances and covariances for each test were adjusted by a heterogeneity factor (Chi-square value (χ^2^) divided by the degrees of freedom (df)), and the critical value from the *t*-distribution was then used to compute the fiducial limits for the LC_50_ and LC_99_ [38]. The calculated LC_50_ values in Table 1 suggest that eggs are the most tolerant life stage for EDN, but only because the LC_50_ for eggs was about twice that for mobile stages of *T. putrescentiae*. Additionally, the percent mortality data in Figure 1 show that 100% mortality was achieved in mobiles at a lower concentration than the eggs.

### 3.2. Mixed Life-Stage Fumigation Results

Fumigation results of mixed life stages are reported in Table 2. Complete control of *T. putrescentiae* was achieved at a fumigation rate of 1.3 mg/L, and less than 0.05% of the population survived after a treatment at the fumigation rate of 0.6 mg/L within a 24 h treatment at 25 °C.

### 3.3. Sorption and Desorption of EDN on Dry-Cured Country Ham

**Figure 2:**
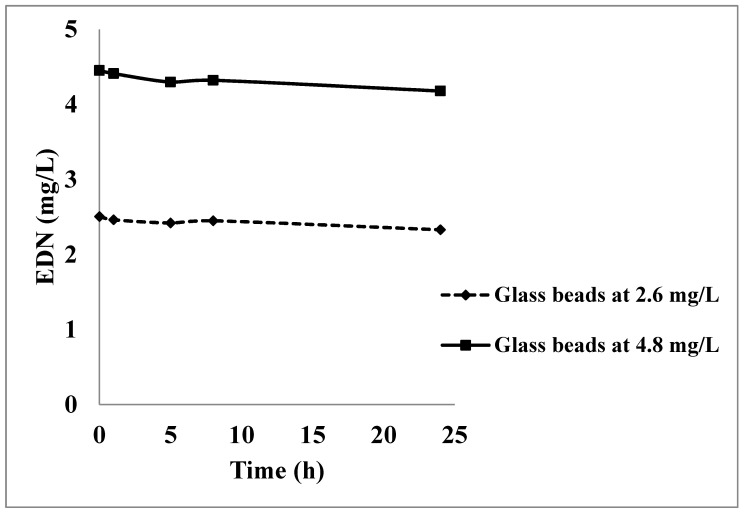
Change in the free-headspace concentration of EDN in the two fumigation chambers containing glass beads over 24 h after application of either 2.6 or 4.8 mg/L at 25 °C.

**Figure 3:**
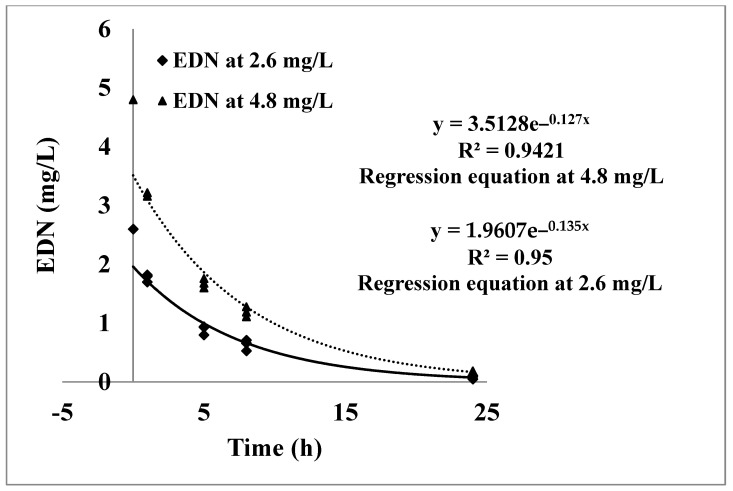
Losses in the free-headspace concentrations of EDN, sorption, in three fumigation chambers during a 24 h fumigation at concentrations of 2.6 and 4.8 mg/L at 25 °C.

The free-headspace concentrations of EDN in the fumigation chambers at concentrations of 2.6 and 4.8 mg/L are shown in Figure 2 and Figure 3, respectively. The results indicated that there was no significant decrease in the free-headspace concentrations of EDN in the fumigation chambers containing the glass beads at both applied concentrations of 2.6 and 4.8 mg/L (Figure 2). On the other hand, there were significant decreases in the free headspace concentration of EDN in the fumigation chambers that contained the ham meat (Figure 3), indicating that the decline in gas concentration was due to the sorption of EDN by the ham meat. The free-headspace concentration of EDN was 1.77 and 3.18 mg/L one hour after application of 2.6 and 4.8 mg/L, which was a 30% decrease in EDN concentration in one hour. After 24 h of fumigation, the free-headspace average concentrations of EDN at application concentrations of 2.6 and 4.8 mg/L dropped to 0.08 and 0.13 mg/L, respectively, indicating that more than 97% of the applied concentrations had declined. Also, the decrease in the free-headspace concentrations of EDN at both application concentrations followed a first-order kinetics equation which can be described as (Ct = 2.085e − 0.137t) with R^2^ of 0.9504 at 2.6 mg/L and (Ct = 3.672e − 0.142t) with R2 of 0.9406 at 4.8 mg/L (Figure 3).

**Figure 4:**
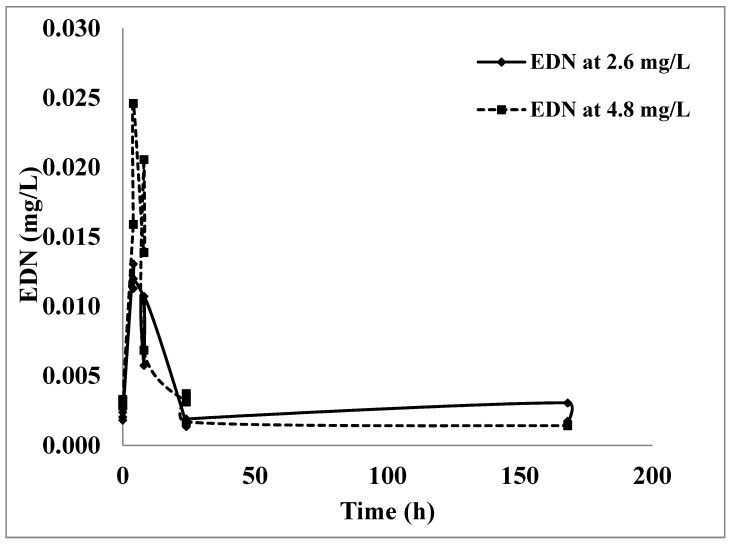
Change in the free-headspace concentrations of EDN (mg/L) in the fumigation chambers during 168 h to allow for desorption at 25 °C. Chambers were originally fumigated for 24 h with EDN at 2.6 and 4.8 mg/L, and then ventilated for 1 h and resealed before samples were taken to determine desorption of EDN.

The ham meat sorbed more than 30% of the initial applied concentrations after 1 h with sorption values of 7.46 and 14.55 mg/kg at 2.6 and 4.8 mg/L, respectively. By the end of 24 h of fumigation, the sorption of EDN by the ham meat was 22.65 and 42.01 mg/kg, indicating that more than 96% of the concentration applied was sorbed. In the desorption trial, the results showed that there was no substantial desorption of EDN released from the ham meat after ventilation of the jars, with approximately 0.009 and 0.014 mg/L after 8 h of desorption from 2.6 and 4.8 mg/L fumigations, respectively, in Figure 4.

## 4. Discussion

Concentration–mortality results show that *T. putrescentiae* eggs (LC_50_ = 0.61 mg/L) were more tolerant to EDN than mobiles (LC_50_ = 0.37 mg/L). The difference between these two LC_50_ values is not substantial since the fiducial limits overlap the calculated LC_50_ value for each, and the fiducial limits for the eggs are acceptable. Prior work in our lab showed eggs of two beetle species studied had much lower LC_50_ values than did adults, with the conclusion that eggs were more susceptible to EDN than adults [39]. The concentration mortality data of EDN did not fit the probit model; we attribute this to the 0.2–0.3 mg/L at which EDN is toxic to *T. putrescentiae* mobiles and eggs, respectively (Figure 1). The narrow range causes difficulties with accurately achieving target gas concentrations to be held for the duration of the exposure period. However, the average emergence of *T. putrescentiae* from a 24 h EDN exposure at 1.3 mg/L, which is equivalent to 600 ppm and 25 °C with a recovery period of 72 h for mobiles and 168 h for eggs, was favorable for complete control of both life stages. Mortality of the mites may be enhanced due to the high relative humidity that is necessary to keep mites from desiccating, and the increased toxicity of EDN applied under high humidities [20,31]. Hooper et al. (2003) reported enhanced mortality of *R. dominica* with an exposure of EDN at 0.33 mg/L for 17 h and 2 h exposure at various humidities [31].

To our knowledge, this is the first study to evaluate EDN’s efficacy against *T. putrescentiae*. Results from our study indicate that *T. putrescentiae* reaches 100% mortality like *T. confusum* at ~300–600 ppm or 0.6–1.3 mg/L [31]. Additionally, studies by Ren et al. [34] reported that EDN was about 4.5 times more toxic than methyl bromide for *T. castaneum* and twice as toxic to *T. variable* and *L. serricorne*. Ramadan et al. [39] reported that EDN exerted high toxicity against all life stages of *L. serricorne* with eggs being the most susceptible stage with an LC_50_ value of 50.4 ppm (0.1 mg/L) followed by adults, pupae, and larvae with LC_50_ values of 160.2 ppm (0.3 mg/L), 192.5 ppm (0.4 mg/L), and 446.6 ppm (1.0 mg/L), respectively. Also, the same group reported that eggs of *R. dominica* (LC_50_ = 11.2 ppm (0.02 mg/L) were more susceptible to EDN than adults at LC_50_ = 27.7 ppm (0.05 mg/L).

We found that EDN is highly sorbed by ham. The initial concentrations applied decreased by 97% after 24 h of fumigation at 25 °C. To the best of our knowledge, no studies have been reported on EDN sorption by ham meat. However, our results are comparable to other studies that reported the EDN sorption by agricultural commodities. For example, Ramadan et al. [41] found that the initial concentrations applied of EDN (2.6 and 4.8 mg/L) decreased by levels of 99.8, 98.9, 98.5, 96.2, and 84.3% for wheat flour, wheat kernels, pinto beans, corn, and tobacco leaves, respectively, after a 24 h fumigation at 25 °C. In addition, the sorption percentage of EDN by timber blocks was reported to be 63% after 48 h of fumigation at 25 °C [39]. Park et al. [42] showed that the initial concentration of EDN applied was decreased by more than 87% after 24 h of log fumigation. Also, the present study showed that the decrease in EDN concentrations with time followed the first-order kinetics, which agreed with previous studies on fumigant sorption by commodities [41,43,44,45]. Given the presence of ham or other sorptive materials, it is important to maintain the EDN concentration at a high enough concentration at the beginning of the fumigation process to keep the gas at an appropriate concentration during the fumigation period to achieve complete control. As for food safety, EDN-treated hams should be studied for EDN residues after treatment to determine if the treated ham is dangerous for human or animal food or if sorbed EDN has broken down into non-toxic reaction products.

Future studies to determine fumigant exposure times under various conditions, quantitative sorption, desorption assays of EDN on selected stored products, and the quantification of any EDN residues will be beneficial before conducting commercial-scale fumigation studies to control *T. putrescentiae*.

## Figures and Tables

**Table 1:** Probit analyses of mortality for *Tyrophagus putrescentiae* life stages to tested concentrations of ethanedinitrile during a 24 h exposure at 25 °C.

Life Stage	n	Slope ± SE	Intercept ± SE	LC_50_ (F.L.) (mg/L)	LC_99_ (F.L.) (mg/L)	χ^2^	DF	*p* > χ^2 a^	R^2^
Egg	608	0.36 ± 0.13	0.52 ± 0.25	0.61 (0.36–19.24)	9.03 (1.71–856,372.72)	108.14	28	<0.01	0.20
Mobile	961	0.78 ± 0.12	1.16 ± 0.25	0.37 (N.A.)	484.20 (N.A.)	16,488.96	22	<0.01	0.64

^a^ If *p* > χ^2^ is larger than 0.05, then there would be a significant fit between the observed data and the expected regression line. As *p* was <0.01, the mortality data did not meet a probit regression.

**Table 2:** The average and standard error of survived mobile life stages at three- and five-days post fumigation with ethanedinitrile (EDN) on *Tyrophagus putrescentiae* mixed life-stage fumigations at concentrations 1×, and 2× the concentrations that caused 100% mortality in concentration–response studies (n = 5).

Recovery Days	Number of Mobile Survivors at Different Concentrations (mg/L) of		
	EDN		
	0	0.6	1.3
3	9758 ± 1170	3.0 ± 0.9	0.0 ± 0.0
7	16,360 ± 771.5	5.0 ± 2.1	0.0 ± 0.0

## Data Availability

The data presented in this study are available on request from the corresponding author. The data are not publicly available due to privacy restrictions on the authors.
